# Risk of Thromboembolic Events in Patients with Non-Valvular Atrial Fibrillation After Dabigatran or Rivaroxaban Discontinuation – Data from the Ljubljana Registry

**DOI:** 10.1371/journal.pone.0156943

**Published:** 2016-06-09

**Authors:** Nina Vene, Alenka Mavri, Mirjam Gubenšek, Gregor Tratar, Tjaša Vižintin Cuderman, Maja Pohar Perme, Aleš Blinc

**Affiliations:** 1 Department of Vascular Diseases, University Medical Centre Ljubljana, Ljubljana, Slovenia; 2 Division of Internal Medicine, Faculty of Medicine, University of Ljubljana, Ljubljana, Slovenia; 3 Institute of Biomedical Informatics, Faculty of Medicine, University of Ljubljana, Ljubljana, Slovenia; IIBB-CSIC-IDIBAPS, SPAIN

## Abstract

**Background and Aim:**

Interruption of anticoagulant treatment with warfarin or non-vitamin K antagonist oral anticoagulants (NOAC) represents a vulnerable period with an increased risk of thromboembolic events. What is the incidence of thromboembolic events in real-life patients with non-valvular atrial fibrillation treated with NOAC who had a discontinuation or cessation of treatment in comparison to patients on continuous treatment?

**Patients and Methods:**

Registry data from 866 patients with non-valvular atrial fibrillation, aged 74.3 (SD 9.8) years, with an average CHADS_2_ score of 2.1 (SD 1.2), who were started on dabigatran or rivaroxaban, were analysed for thromboembolic events and survival. Patients who had temporary or permanent discontinuation of NOAC were compared to patients on continuous NOAC treatment.

**Results:**

Among 866 patients started on NOAC, 705 were treated without interruption, 84 patients had temporary interruption (69 because of planned invasive procedures, 10 due to bleeding, 5 for other causes) and 77 had permanent cessation of NOAC treatment. In patients without interruptions, the incidence of thromboembolic events was 1.0 (95% CI 0.4–2.1) per 100 patient-years, while in patients with interruption/cessation the rate of thromboembolic events was 21.6 (95% CI 10.3–45.2) per 100 patient-years, p < 0.001. There was a distinct clustering of thromboembolic events in the first weeks of NOAC discontinuation with the median occurring on day 14 (range 1–37 days) after discontinuation.

**Conclusion:**

Dabigatran and rivaroxaban offered good protection against thromboembolic events during treatment, but interruption of NOAC treatment increased the short-term thromboembolic risk more than 20-fold.

## Introduction

Dabigatran, an oral thrombin inhibitor, and rivaroxaban, an oral factor Xa inhibitor, are at least non-inferior to warfarin in reducing the risk of non-valvular atrial fibrillation (AF)-related thromboembolic complications [1, 2]. The main advantages of non-vitamin K antagonist oral anticoagulants (NOAC) over warfarin are their rapid and predictable onset of action, fixed-dosing, few food and drug interactions and no need for routine anticoagulant intensity monitoring [3]. The half-life of NOAC is short compared to warfarin, therefore interruption of treatment results in rapid reversal of the anticoagulant effect [3].

Temporary interruptions of oral anticoagulant treatment occur most often due to planned invasive procedures or bleeding. Interruptions of warfarin treatment in patients with AF are associated with highly increased short-term risk of death or thromboembolic events [4]. In clinical trials, a similarly increased risk of stroke was observed following discontinuation of rivaroxaban or apixaban [5–7]. Data is emerging that discontinuation because of bleeding is much more dangerous [8–12] than discontinuation due to planned invasive procedures [13–16]. However, apart from case reports [17,18] and data from the Dresden registry [19] there has been no systematic analysis of the thromboembolic risk after NOAC discontinuation in real-life practice.

Our aim was to analyse the real-life incidence of thromboembolic events following interruption or cessation of dabigatran or rivaroxaban in comparison to patients on continuous treatment with NOAC. Additionally, among patients with interruptions/cessations, we compared bleeding episodes to planned invasive procedures.

## Patients and Methods

### Patients and follow-up

At the Anticoagulation Clinic of the Department of Vascular Diseases, University Medical Centre Ljubljana, Slovenia, serving a region with about 700,000 inhabitants, we have been running a registry of outpatients on anticoagulation treatment in order to systematically evaluate the efficacy and safety of treatment (Trombo registry). The University Medical Centre Ljubljana registry of patients on anticoagulation drugs and this study has been approved by the Committee for Medical Ethics of the Republic of Slovenia with their decision letter No. 150/10/12 of November 24, 2012. Since all patients included in the registry received routine clinical care according to current standards of best medical practice and no experimental intervention was part of this study, all patients consented to participating in the registry verbally and their consent was recorded in their file. The Committee for Medical Ethics of the Republic of Slovenia agreed with obtaining consent verbally, provided that the patients’ data was stored securely and that patients’ privacy was respected at all times when analysing the registry data. The patient data has been managed securely by the computer program Trombo (Magas d.o.o., Slovenia), designed for recording and analysing patient characteristics and adverse events as well as for assisting in treatment-related decisions.

Between February 2012 and December 2013, a total of 2443 patients with AF were referred to our anticoagulation clinic for initiating anticoagulation in order to prevent stroke. Among those, 866 patients with non-valvular AF, i.e., AF not associated with artificial heart valves or mitral stenosis, were started on NOAC: 549 patients on dabigatran (110 or 150 mg twice daily) and 317 patients on rivaroxaban (20 or 15 mg once daily). The lower dose of NOAC, i.e. dabigatran 110 mg twice daily or rivaroxaban 15 mg daily, was prescribed to patients with moderately impaired renal function (estimated glomerular filtration rate 30–50 ml/min), elderly patients (>75 years), those with a history of major bleeding, those on amiodarone, verapamil or antiplatelet drugs, and to frail patients, a total of 328 patients received the lower dose dabigatran and 86 patients the lower dose rivaroxaban. The baseline characteristics of patients started on NOAC are shown in Table 1. The average CHADS_2_ score was 2.1±1.2 and the average HASBLED score was 1.0±0.6.

**Table 1:** Characteristics of the patients started on NOAC.

Characteristics of patients (n = 866)	
Age [years (SD)]	74.3 (9.8)
Gender [male/female (%)]	456/410 (52.7/47.3)
CHADS2 score [n (%)]	
0–1	320 (37.0)
2	294 (33.9)
≥3	252 (29.1)
HASBLED [n (%)]	
0–1	545 (62.9)
2–3	258 (29.8)
>3	63 (7.3)
CONCOMMITANT CONDITIONS [n (%)]	
Arterial hypertension	727 (83.9)
Heart failure	139 (16.0)
Diabetes mellitus	165 (19.0)
Previous stroke or TIA	173 (20.0)
Renal function impairment (eGFR 30–60 ml/mn)	87 (10.0)
Concommitant use of antiplatelet drugs (aspirin)	29 (3.3)
Type of atrial fibrillation:paroxysmal/ permanent	317 / 549 (36.6/ 63.4)

The patients’ thromboembolic risk has been evaluated by the CHADS_2_ score and the bleeding risk by the HAS-BLED score [20, 21]. Before starting NOAC treatment, renal and liver function, haemoglobin and platelet count were assessed in all candidates. As a surrogate measure of renal function we used the estimated glomerular filtration rate (eGFR) calculated by the Modified Diet in Renal Disease (MDRD) equation. Patients were eligible for NOAC treatment if the liver enzymes and bilirubin were ≤ 2-times the upper limit of normal, the haemoglobin level ≥ 100 g/l, platelets ≥ 100 x 10^9^/l and eGFR ≥ 30 ml/min. Patients receiving NOAC were scheduled for follow-up visits at 1, 3–6 and 12 months.

Thromboembolic, haemorrhagic or other adverse events were documented during the regular visits. Thromboembolic events comprised a documented ischemic stroke or transient ischemic attack, acute coronary syndrome, and thromboembolism affecting visceral organs or limbs. Major bleeding was defined according to the ISTH criteria–in brief, as a fatal bleed, bleeding into vital organs, or acute reduction of haemoglobin by > 20 g/l [22]. For patients who missed their scheduled appointments, as well as for patients after treatment cessation, we made telephone inquiries with patients, their relatives or primary care physicians in order to document all adverse events and deaths in our cohort.

### Statistical analysis

Descriptive statistics for numerical variables are reported as means and standard deviations or in case of asymmetric distributions, as medians and ranges. Categorical values are reported with group frequencies and percentages.

The total number of person-years under observation is reported when reporting duration.

The incidence of thromboembolic events has been calculated as the number of events per 100 patient-years (pty) of discontinued NOAC treatment or per 1 pty of discontinued NOAC treatment for subgroup analysis, with the 95% confidence interval based on the Poisson distribution. The observed incidence of thromboembolic events during interruption /discontinuation of NOAC treatment has been compared to the expected incidence of thromboembolic events based on each patient’s CHADS_2_ score and length of observation.

The event free survival has been calculated by the Kaplan-Meier method and compared between subgroups by the log-rank test. In addition, we have fitted a piecewise exponential model to compare the hazard of events in the first 30 days of interruption to the hazard at longer times.

## Results

### Continuous treatment vs. cessation or interruption of NOAC

**Fig 1:**
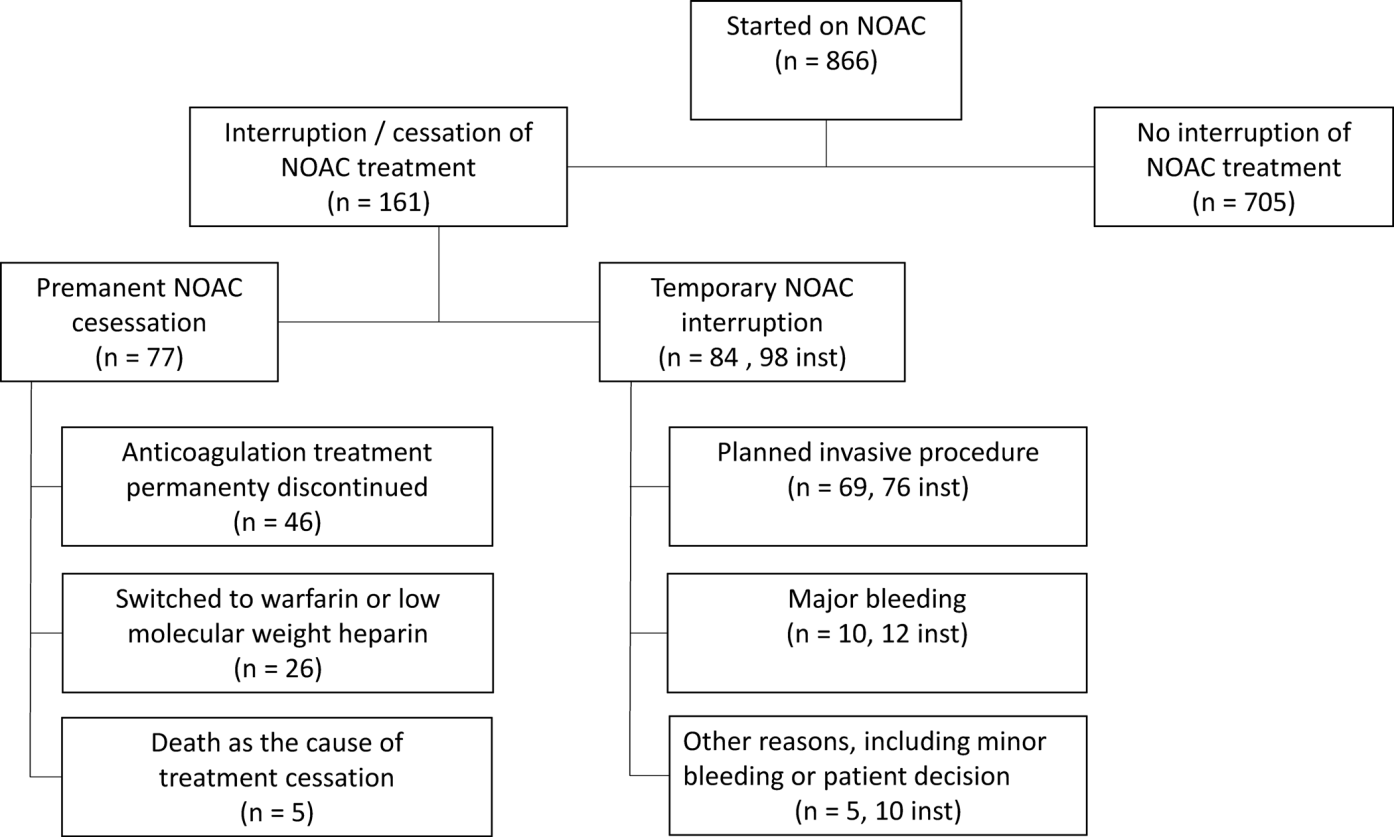
The fate of patients stared on non-vitamin K oral anticoagulants (NOAC) regarding interruption or cessation of NOAC treatment. (n–number of patients, inst–number of instances).

The fate of our patients regarding cessation or interruption of treatment is shown in Fig 1. The average duration of follow-up for all patients was 9±4 months. In 705 patients there was no interruption of anticoagulant treatment with NOAC, while in 161 patients treatment with NOAC was interrupted or stopped.

**Fig 2:**
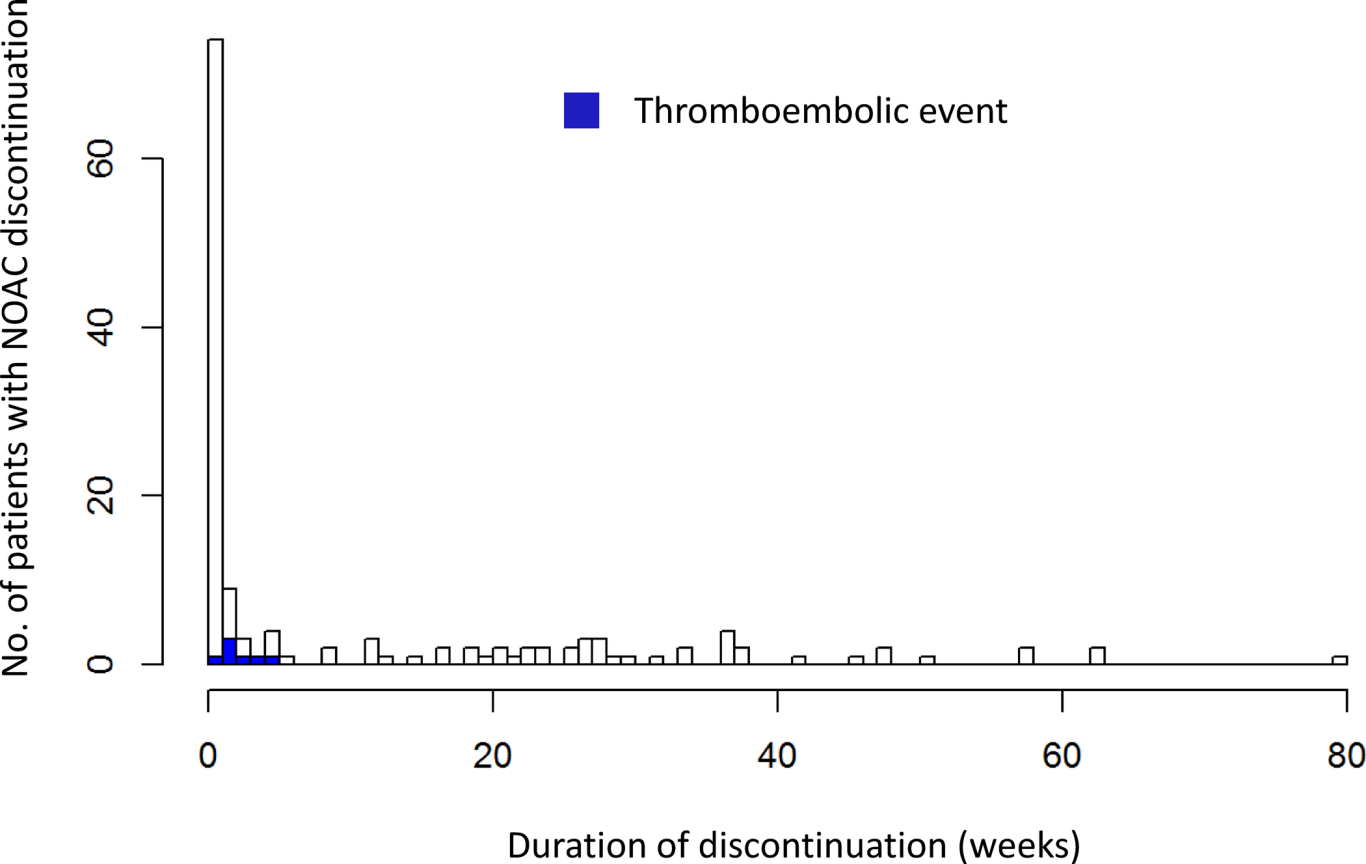
The number of patients with NOAC discontinuation as a function of the duration of the discontinuation. The dark coloured rectangles denote the instances of thromboembolic events. In this series, no patient suffered more than one thromboembolic event. Most interruptions lasted less than 1 week and thromboembolic events distinctly clustered in the first 4 weeks after discontinuation.

Among 84 patients there were 98 temporary treatment interruptions with a median duration of 7 days (Fig 2). The reasons for temporary interruptions were: planned invasive procedures in 69 patients (76 instances, 51% of discontinuations) with a median duration of peri-procedural interruption 4 (range 2–15) days, major bleeding in 10 patients (12 instances, 8% of discontinuations) with a median duration of interruption 17 (range 6–33) days, and other reasons, like dyspepsia, patient decision or minor bleeding in 5 patients (10 instances, 7%) with a median duration of 37 (range 21–209) days. Patients with treatment interruptions were followed for a median 6.6 (range 0.1–19.9) months.

Anticoagulation treatment was intentionally stopped in 46 patients on NOAC (34% of all anticoagulant treatment discontinuations), 1–2 months after successful conversion of atrial fibrillation into sinus rhythm in patients with a low thromboembolic risk and a CHADS_2_ score of 0–1. For 5 patients, death was the cause of permanent discontinuation of NOAC treatment (1 death due to ischemic stroke, 1 due to myocardial infarction, 3 due to non-cardiovascular cause). An additional 26 patients were switched from NOAC to long-term warfarin because of worsening renal function, or to low molecular weight heparin because of newly diagnosed malignancies.

The comparison in baseline characteristics between patients on continuous NOAC in comparison with patients who had interruptions or cessation showed that the former were on average 2 years older—age (74.7 (SD 9.4) vs. 72.6 (SD 11.4) years, p = 0.011), but there were no significant differences in the proportion of females (48.5 vs. 42.2%, p = 0.19), proportion of HAS BLED score of ≥ 2 (37.0% vs. 37.3%, p = 0.90), or distribution of CHADS_2_ scores (CHADS_2_ 0–1 points 36.3% vs. 39.8%, CHADS_2_ 2 points 34.2% vs. 31.7%, CHADS_2_ ≥ 3 points 29.5% vs. 28.5%, overall p = 0.558).

### Thromboembolic events

Seven thromboembolic events (95% CI 3.3–14.7 events) occurred during NOAC interruption/cessation among 161 patients with a cumulative follow-up of 32.5 patient years of observation (pty), while 6 (95% CI 2.7–13.4) thromboembolic events occurred during uninterrupted NOAC treatment among 705 patients with a cumulative follow-up of 621.7 pty. The incidence of thromboembolic events in patients with interruption/cessation of any cause was thus 21.6 (95% CI 10.3–45.2) per 100 pty, while in patients without interruptions it was 1.0 (95% CI 0.4–2.1) per 100 pty, p < 0.001.

**Fig 3:**
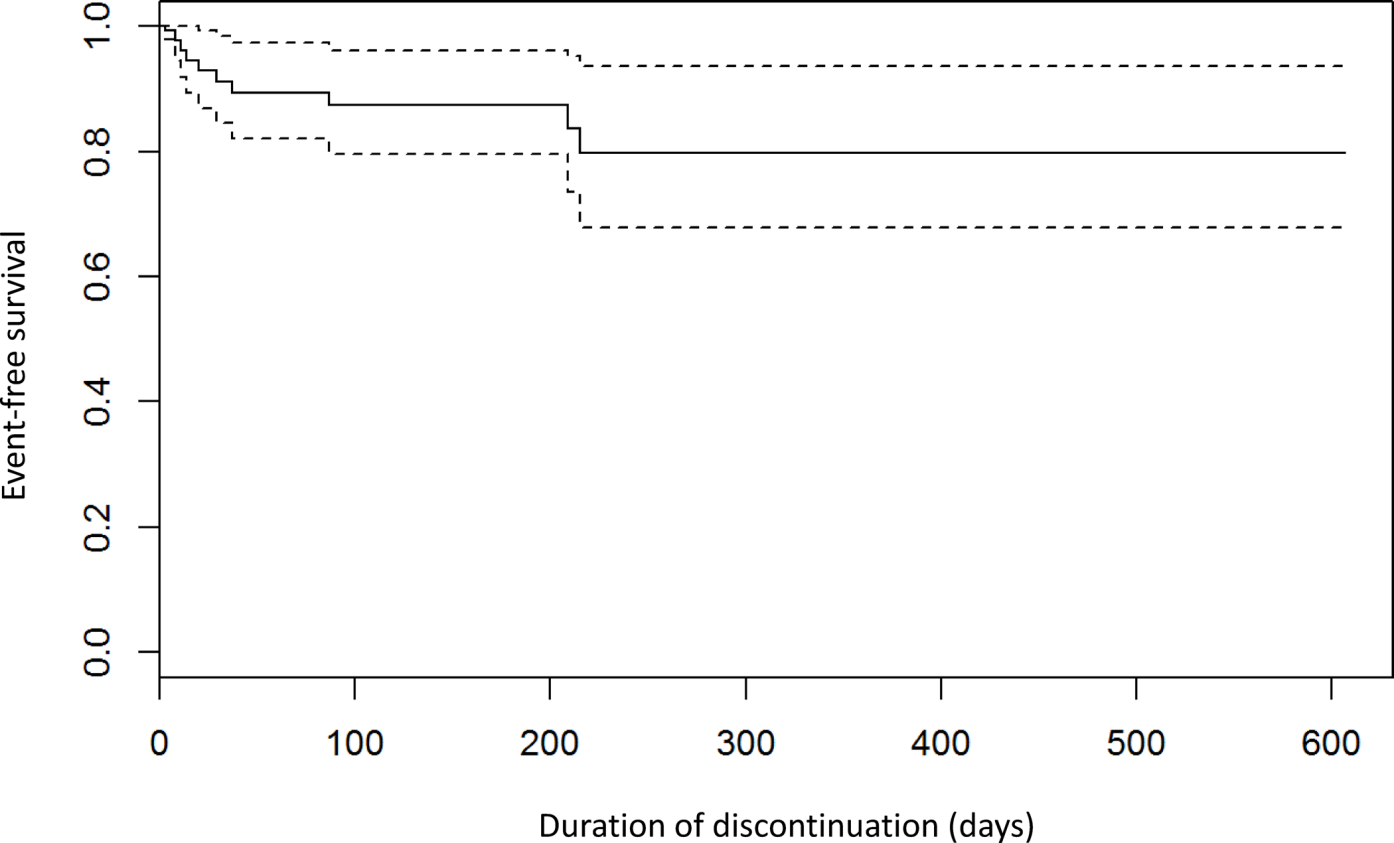
Probability of survival without thromboembolic events or all-cause death for patients who discontinued treatment with NOAC. Time 0 is the time of the interruption/cessation. The 95% confidence intervals are marked with interrupted lines. All non-fatal thromboembolic events occurred within 37 days after interruption/cessation.

The observed incidence of thromboembolic events during interruptions of treatment was significantly higher than the predicted “natural-course” incidence based on the CHADS_2_ scores and lengths of observation (Table 2). On the other hand, in patients with uninterrupted NOAC treatment the incidence of thromboembolism was significantly lower than the incidence predicted by the CHADS_2_ score for untreated patients (Table 2). The piecewise exponential model for clustering of events showed a distinctly different coefficient in the first 30 days of interruption compared longer interruptions. The estimated hazard of a thromboembolic event during the first 30 days was 6.9-times higher than beyond 30 days (p = 0.0013) (Fig 2). Event-free survival of patitents with NOAC discontinuation is shown in Fig 3.

**Table 2:** Observed incidence of thromboembolic events during interrupted and uninterrupted NOAC treatment and comparison with the expected incidence based on the CHADS_2_ score (20) and length of observation if patients were left untreated. The 95% confidence intervals are given in brackets. The p values were calculated according to the Poisson model. NS–non-significant.

	Uninterrupted treatment	Interruption / cessation of treatment	p-value
**Observed** incidence of thromboembolic events, per 100 pty	1.0 (0.4–2.1)	21.6 (10.3–45.2)	< 0.001
Expected* incidence of thromboembolic events if left untreated, per 100 pty	4.6 (3.4–6.0)	3.3 (2.4–4.4)	NS
p-value	< 0.01	< 0.001	

*expected incidence based on the CHADS_2_ score (20) and lengths of observation

### Subgroup analysis

#### Type of AF

Two thromboembolic events occurred during interruption of NOAC treatment among patients with persistent / chronic AF and 5 events occurred among 64 patients with paroxysmal AF. However, we could not prove a difference in the incidence of thromboembolic events between patients with persistent/chronic or paroxysmal AF: 12.2 (95%CI 3.2–51.6) events per 100 pty vs. 31.0 events (95%CI 13.0–78.2) per 100 pty, p = 0.269.

#### Interruption because of bleeding vs. planned invasive procedure

In the subgroup of patients with an interruption due to bleeding there were 4 thromboembolic events among 10 patients with a cumulative follow up of 0.59 pty, while for planned interruptions due to invasive procedures there was only 1 event among 69 patients with a cumulative follow up of 0.93 pty. The proportion of thromboembolic events during NOAC interruption among bleeders was significantly higher than in patients with interruptions due to planned invasive procedures (p < 0.001 by Fisher’s exact test). However, when taking into account the longer duration of NOAC interruptions among bleeders in comparison to the short interruptions of planned invasive procedures, the difference in incidence according to the Cox model was not significant (6.7 (95% CI 2.5–17.9) per pty vs. 1.1 (95% CI 0.15–7.6) per pty, p = 0.40). No significant differences were found among bleeders and patients with interruptions due to invasive procedures in terms of age (74.4 (SD 6.7) vs. 71.0 (SD 10.8) years, p = 0.2), proportion of females (30.0 vs. 40.3%, p = 0.73), proportion of HAS BLED of ≥ 2 (20.0% vs. 29.0%, p = 0.71), or distribution of CHADS_2_ scores (CHADS_2_ 0–1 points 50.0% vs. 47.0%, CHADS_2_ 2 points 20.0% vs. 30.5%, CHADS_2_ ≥ 3 points 30.0% vs. 22.5%, overall p = 0.82).

## Discussion

Our analysis confirmed that NOAC offered good protection against thromboembolic events in real-life patients with non-valvular AF, but that interruptions of treatment represented a vulnerable period, especially when they were caused by bleeding.

The 1% annual incidence of ischemic stroke or embolism in our patients on uninterrupted NOAC treatment was similar to the published data from the RELY and ROCKET studies [1, 2] which is more than 4-fold lower than the CHADS_2_ score-predicted incidence if the patients were left untreated. In contrast, the incidence of thromboembolic events during treatment interruptions was more than 6-times higher than the “natural course” incidence predicted by the CHADS_2_ score. Furthermore, during the first 30 days after discontinuation of NOAC the hazard of a thromboembolic event in the first 30 day was 6.9-times higher than during the second and following months of discontinuation, when the hazard basically returned to the CHADS2 score- predicted level. This raises the possibility of a thrombogenic effect of the reason for discontinuation or a rebound hypercoagulability effect shortly after discontinuation.

The proportion of patients who suffered a thromboembolic event after NOAC discontinuation was higher among bleeders than among patients with planned invasive procedures. However, there was no significant difference in the incidence of thromboembolism, because interruptions due to bleeding took longer and “diluted” the effect of the vastly different proportion of thromboembolic events among patients: 4/10 with major bleeding vs. 1/69 with planned invasive procedures. The different proportions of patients suffering a thromboembolic event after NOAC discontinuation because of bleeding or because of planned invasive procedures could not be explained by different baseline risk, since we found no significant differences in age, proportion of females, HAS BLED score ≥ 2 or distribution of CHADS_2_ scores. The association between bleeding and increased thrombotic risk is biologically plausible, since vascular injury activates platelets and coagulation. High rates of thromboembolism have been reported within 30 days of stopping warfarin because of gastrointestinal bleeding [8, 9]. In patients with acute coronary syndrome, bleeding has been associated with an increased risk of subsequent myocardial infarction and stroke for a month after the bleeding event [10, 11]. Major bleeding in patients with atrial fibrillation who were treated with apixaban or warfarin in the ARISTOTLE trial increased the risk of death, ischemic stroke or myocardial infarction 12-fold in the first month after the bleed [12]. It is becoming clear that an increased risk of thromboembolic events after bleeding is a universal phenomenon with all anticoagulation drugs [8–12].

Planned invasive procedures, the most common causes of temporary interruptions of NOAC treatment, were short, with a mean duration of only 4 days. The observed incidence of thromboembolic events of 1.1 (95% CI 0.15–7.6) per pty is consistent with observations of other authors who report low risk (around 1%) of post-procedural stroke, systemic embolism or cardiovascular events during 30-day follow up after brief interruptions of NOAC or warfarin treatment due to invasive procedures [6, 13–16, 19, 23], indicating that short periprocedural interruptions of any type of oral anticoagulation in daily care are safe.

As for the potential rebound effect after discontinuing NOAC, it has been reported that thrombin generation is increased in the presence of low concentrations of dabigatran, while no such effect has been noted with rivaroxaban [24]. A hint that rebound hypecoagulability might be among the reasons for increased thromboembolic risk after NOAC discontinuation was given by one of our patients who had a very low stroke risk (CHADS_2_ of 0 and CHA_2_DS_2_-VASc of 0) and no obvious inducers of a hypercoagulable state, but suffered an ischemic stroke ten days after dabigatran discontinuation.

### Study limitations

Due to the relatively low number of events the data were not analysed separately for dabigatran and rivaroxaban and no distinction can be made between these two NOACs. For the same reason it was not possible to analyse the influence of different CHADS_2_ levels on the frequency of thromboembolic events. Our data analysis was simplified by assuming that all interruption intervals were independent. In fact, 6 patients had 2 interruptions and 4 patients had 3 interruptions. Since all thromboembolic events occurred during the first interruption, limiting the analysis only to the first interruption interval would yield a slightly higher, but overall very similar incidence of thromboembolism during interruptions: 22.7 (10.8–47.6) per 100 pty vs. 21.6 (10.3–45.2) per 100 pty.

### Conclusion

Dabigatran and rivaroxaban offer good protection against thromboembolic events during treatment, but discontinuation of NOAC increases thromboembolic risk more than 20-fold, especially in the first month. The proportion of patients who suffered a thromboembolic event was higher among patients with interruptions due to bleeding in comparison to patients with interruptions due to planned invasive procedures.
